# THE CLOSURE OF RURAL CO-HOUSING AND CHANGES IN LONELINESS IN OLDER WOMEN LIVING ALONE

**DOI:** 10.1093/geroni/igae098.1330

**Published:** 2024-12-31

**Authors:** Sojung Park, Soobin Park, Hyunjoo Lee

**Affiliations:** Washington University in St. Louis, St. Louis, Missouri, United States; Washington University in St. Louis, St. Louis, Missouri, United States; Daegu University, Daegu, Republic of Korea

## Abstract

Prolonged social restrictions during the pandemic have increased loneliness among the population, with people living alone in rural areas being particularly vulnerable due to geographic distances, transportation, and finances. Congregate senior housing may be a helpful alternative living environment that can help address the challenges of social isolation. Drawing on the environmental perspective of aging and the literature on loneliness, we investigated the influence of the pandemic-imposed closure of senior cohousing on the changes in loneliness in older women living alone in Korea. Using a mixed-method approach, we analyzed survey data from 160 participants using structural equation analysis focusing on multiple mediators, such as physical, social, and service environment and depressive symptoms. Also, we conducted a thematic analysis with 18 former cohousing residents. Our synthetic analyses showed that senior cohousing protects against loneliness by serving as a community hub for gathering, sharing, and caring among the housing residents and the members of the whole community. We found that community service plays a critical role in protecting against depressive symptoms and loneliness. Our study setting is a publicly subsidized cohousing program with no embedded program manager. The residents are not necessarily empowered yet to self-organize for online technology learning. Intentional programming that includes social activity and online learning would benefit vulnerable subgroups of older women. Our findings make a significant contribution to the limited knowledge base on the intersection of senior housing and rural aging during the pandemic.

